# Ectopic extramammary Paget disease of the scalp following multifocal inguinal disease

**DOI:** 10.1016/j.jdcr.2026.09.004

**Published:** 2026-09-10

**Authors:** Mariana Chrispim Gimenez, Sidra Deen, William Guo, Irina Lerman, Franki Lambert Smith

**Affiliations:** a University of Rochester School of Medicine and Dentistry, Rochester, New York; b Department of Dermatology, University of Rochester Medical Center, Rochester, New York

**Keywords:** ectopic extramammary Paget disease, extramammary Paget disease, Mohs micrographic surgery, scalp extramammary Paget disease

## Introduction

Extramammary Paget disease (EMPD) is an intraepidermal adenocarcinoma that tends to occur in areas with high density of apocrine glands, including the anogenital and axillary skin.^1^^,^^2^ Rarely, EMPD develops in areas with relatively sparse apocrine glands, termed “ectopic EMPD.” Clinically, it typically presents as a scaly, erythematous plaque and may mimic inflammatory, infectious, and neoplastic dermatoses, often contributing to delayed diagnosis.^1^^,^^2^ We report on a 66-year-old male with prior EMPD involving the bilateral inguinal folds who developed ectopic EMPD of the scalp.

## Case report

A 66-year-old male with Fitzpatrick skin type III presented to the Mohs Micrographic Surgery (MMS) department with biopsy-confirmed recurrence of EMPD involving the left inguinal fold. The patient had no significant comorbid conditions, immunosuppressive medication use, or a personal or family history of skin cancer. One year earlier, he had undergone MMS without immunohistochemistry (IHC) to noncontiguous sites of the bilateral inguinal folds for lesions present for approximately 5 years. Concurrent malignancy screening, including urine cytology, colonoscopy, and prostate specific antigen testing, were all unremarkable. Approximately 6 months after surgery, the patient noted a new suspicious lesion inferior to the prior left-sided surgical site. Biopsy confirmed a third distinct site of EMPD.

Upon presentation, the patient underwent several scouting biopsies of the bilateral inguinal folds with IHC staining for cytokeratin 7 (CK7) to delineate subclinical disease (Fig 1). Pathology demonstrated bilateral recurrence and subclinical marginal involvement of all 3 sites. Additional scouting biopsies were subsequently performed and did not reveal further EMPD extension. He returned to clinic for MMS with intraoperative CK7 immunostaining. The tumors were divided into 3 noncontiguous MMS sites, each treated on separate operative days over approximately 2 months. Negative margins were achieved at all sites without marginal overlap. Postoperative healing was uncomplicated, with no adverse events. There has been no evidence of recurrence since the last MMS procedure, approximately 9 months ago.

**Fig 1:**
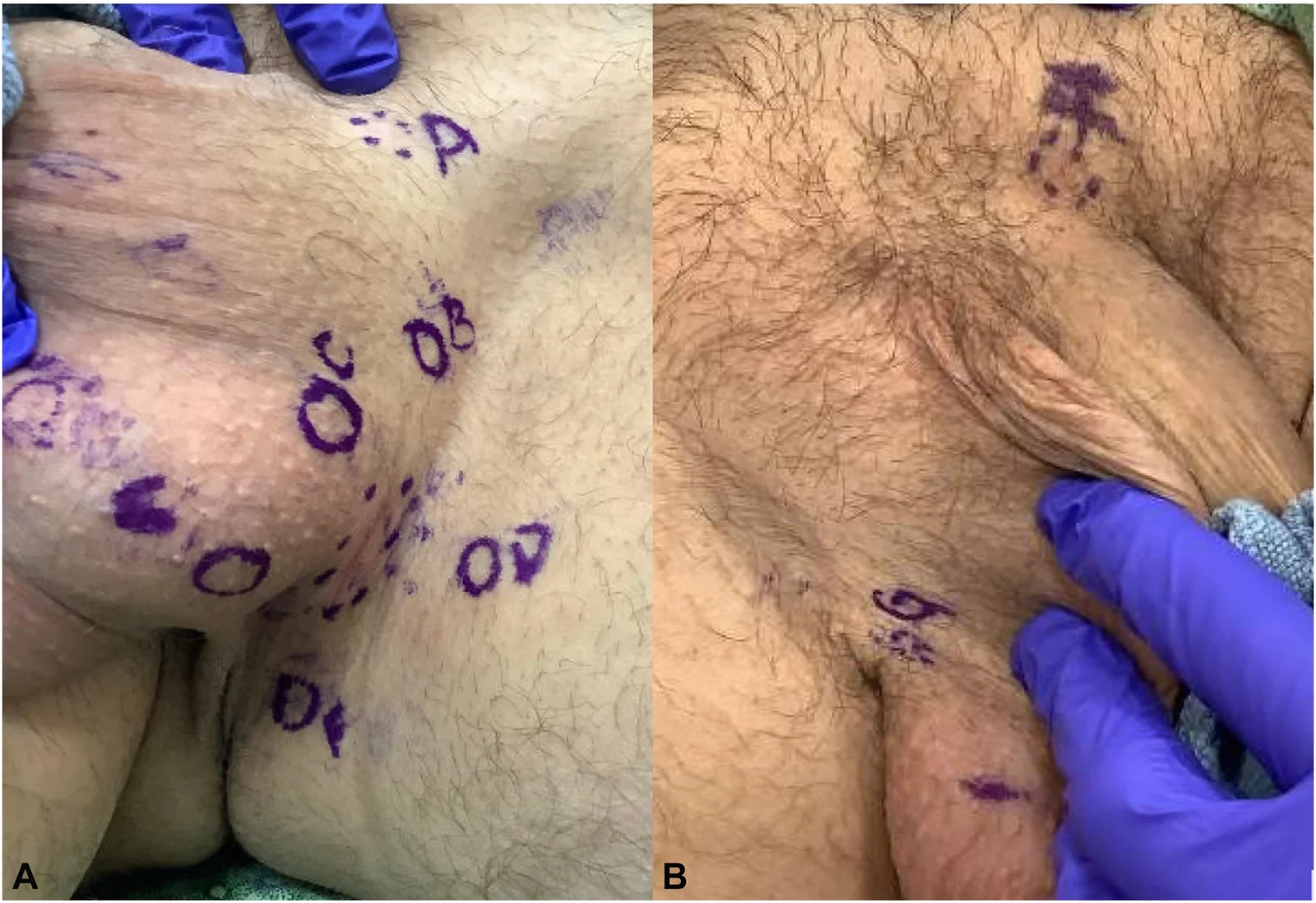
**A,** EMPD site on left inguinal region and scouting biopsy sites. **B,** Scouting biopsy sites on right inguinal region.

Three months following completion of MMS and confirmation of local control, the patient presented to general dermatology for a routine full-body skin examination. He reported a 2.5 × 2 cm intermittently pruritic scalp plaque present for approximately 2 years that had been treated with topical clobetasol without improvement (Fig 2, *A*). Examination of the left parietal scalp revealed an erythematous, mildly scaly plaque, without regional lymphadenopathy. Punch biopsy demonstrated ectopic EMPD extending to the peripheral margins. Stains were positive for CK7 and negative for SOX10 and CK20.

**Fig 2:**
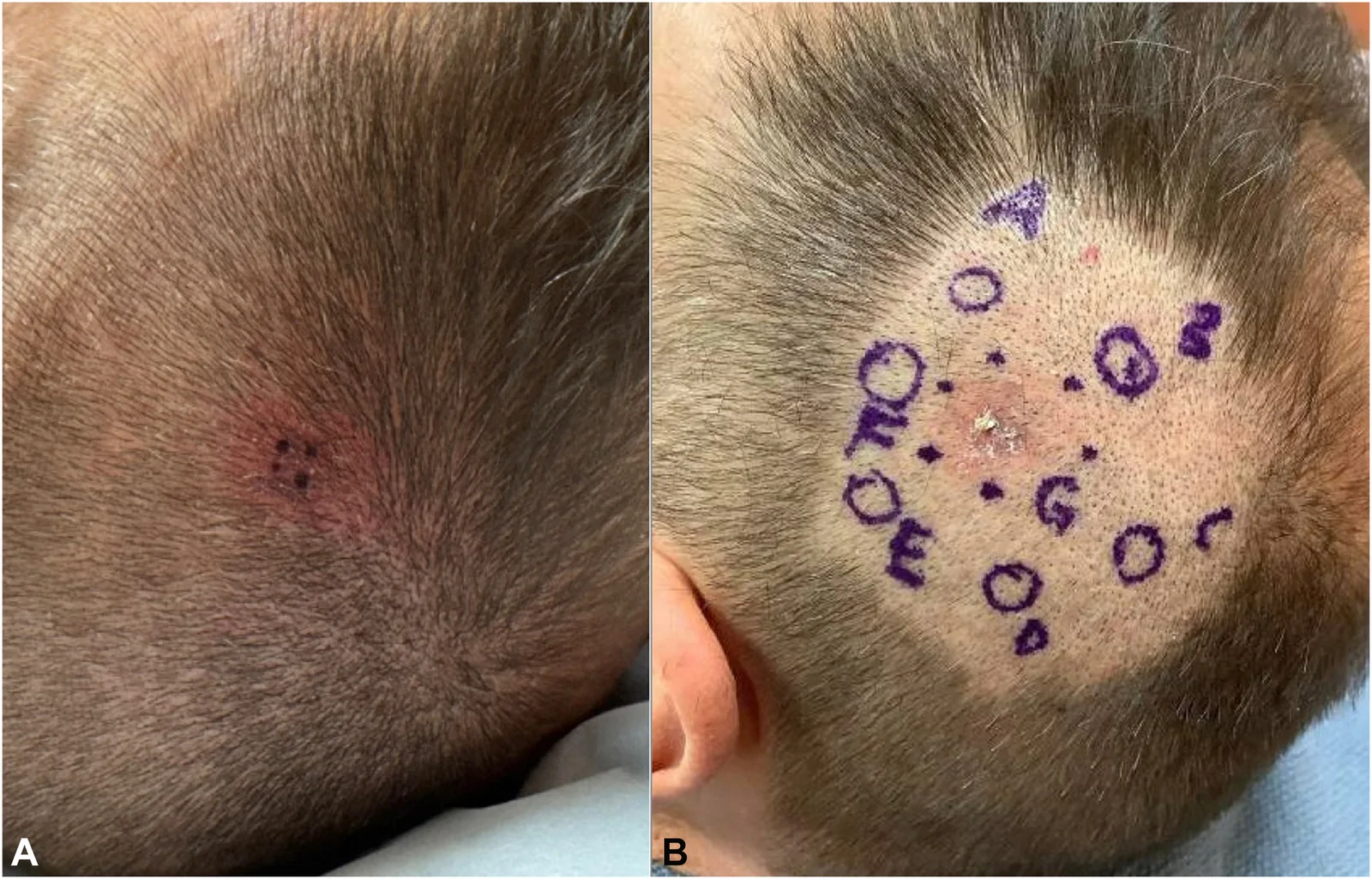
**A,** 2.5 × 2 cm intermittently pruritic plaque on the left parietal scalp. **B,** Ectopic EMPD site on left parietal scalp and scouting biopsy sites.

He was referred again for MMS, and additional scouting biopsies and central debulk were performed by the Mohs surgeon (Fig 2, *B*). One of six scouting biopsies (site E) showed subclinical marginal involvement and a central debulking biopsy showed focally invasive disease extending to a depth of 0.2 mm (Fig 3). Given a prior report of scalp EMPD associated with an adjacent meningioma, magnetic resonance imaging of the head with and without contrast was performed and showed no evidence of malignancy.^2^

**Fig 3:**
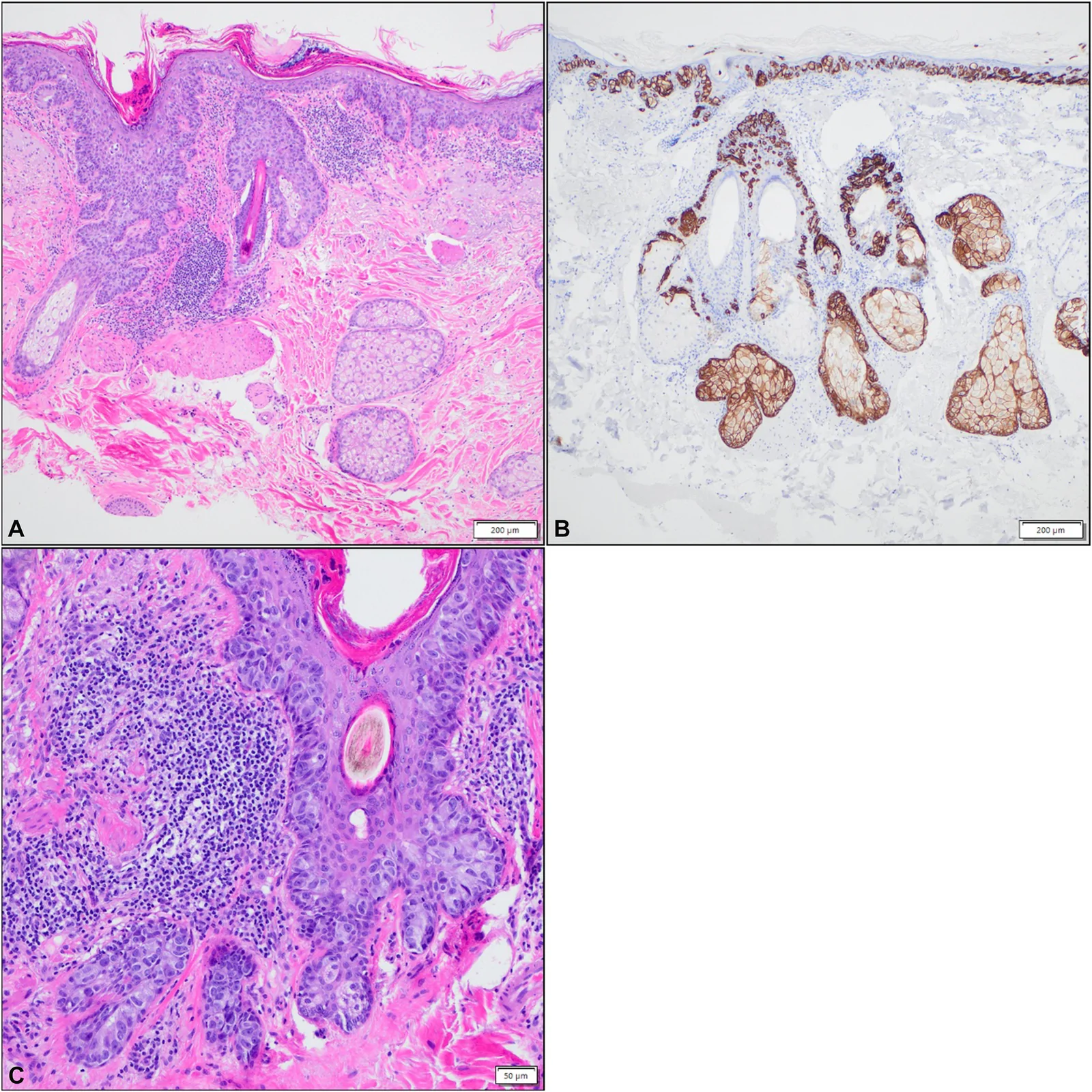
**A,** hematoxylin- and eosin-stained biopsy of central debulk at 40× magnification. **B,** CK7-stained biopsy of central debulk at 40× magnification demonstrating prominent adnexal extension. **C,** Hematoxylin- and eosin-stained biopsy of central debulk at 100× magnification demonstrating small focus of dermal invasion (0.2 mm).

The lesion was treated with MMS with CK7 IHC. Two stages were required to achieve clear microscopic margins, resulting in a 7.3 × 3.5 cm defect repaired with partial primary closure with an area of second intention healing (Fig 4). The patient will continue clinical monitoring and undergo malignancy screenings per guidelines.^3^

**Fig 4:**
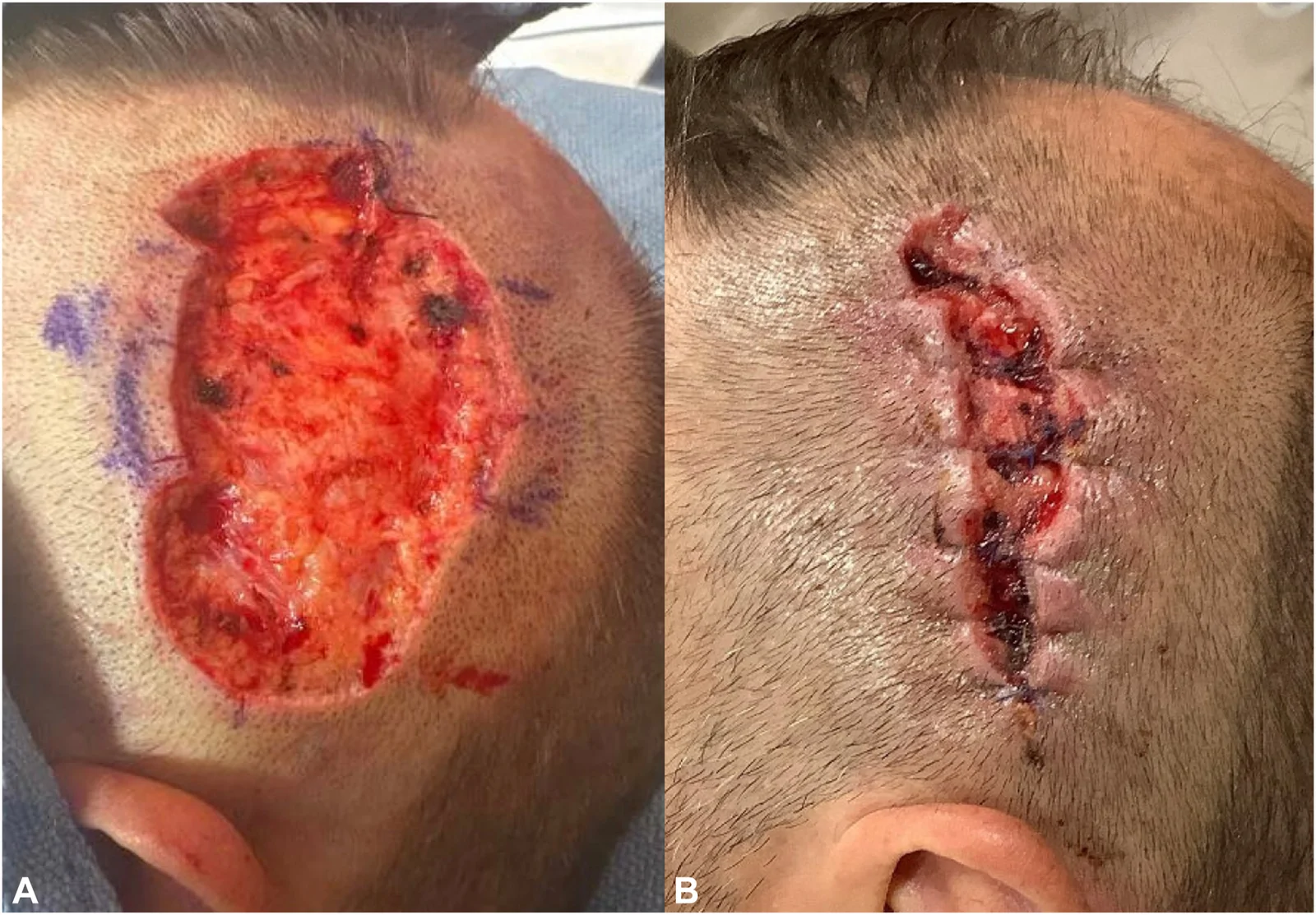
**A,** Final MMS defect. **B,** 2 weeks after simple partial primary repair.

## Discussion

EMPD can be classified as primary, as in this patient with negative screening and imaging tests for internal neoplasms, or secondary, developing through spread of genitourinary and gastrointestinal carcinomas.^1^^,^^4^ EMPD can present as unifocal or multifocal lesions. Some authors believe that EMPD presents as unifocal disease that grows in an irregular growth pattern with areas of subclinical disease.^5^ However, our patient had multifocal disease, as evidenced by the 3 noncontiguous surgical sites cleared with MMS. A small number of cases report EMPD occurring in areas with a low density of apocrine glands, such as the scalp, termed ectopic EMPD.^1^^,^^2^^,^^6^^,^^7^ However, these patients developed ectopic EMPD in the absence of a tumor in apocrine areas, illustrating the unusual nature of the presentation described in this report.^7^

On histopathology, both EMPD and ectopic EMPD are characterized by Paget cells in the epidermis, which are atypical large cells with clear, abundant, and occasionally eosinophilic cytoplasm on hematoxylin and eosin staining.^2^^,^^4^ Paget cells can be found in isolation or in nests.^2^^,^^4^ The histology of EMPD can be resemble pagetoid squamous cell carcinoma in situ and melanoma, highlighting the importance of IHC in diagnosis.^2^^,^^4^ CK7 is an important immunohistochemical marker in the diagnosis of EMPD, as used in the reported case, while CK20 positivity can be associated with secondary EMPD.^2^ In the absence of evidence for or against an associated internal malignancy, additional markers such as TRPS1, SATB2, GATA3, and PSA can be beneficial in differentiating primary and secondary EMPD.^8^

Management of anogenital EMPD is primarily guided by retrospective studies and recently updated treatment and screening guidelines.^2^^,^^3^^,^^9^ Surgery is the first-line option, with topical imiquimod, radiation, and photodynamic therapy serving as second-line options.^8^ MMS has been shown to offer the highest cure rate, with recurrence rates of 11.2% compared to 37% with wide local excision.^9^ Screening guidelines recommend gastrointestinal/genitourinary malignancy screening to distinguish primary from secondary origin.^3^ For ectopic EMPD, therapeutic approaches depend on tumor location and size. Interventions described include surgical excision and radiation.^1^^,^^4^^,^^6^ For our patient who had previously tolerated surgery well, MMS with IHC was selected to offer the highest cure rate.^9^

This case demonstrates a rare occurrence of multifocal EMPD along with ectopic EMPD. Although EMPD classically arises in apocrine-rich regions, this report underscores that disease may extend to or present in atypical, apocrine-poor sites such as the scalp, further complicating recognition. Given its ability to mimic diverse conditions, a high index of suspicion is essential, and histopathologic evaluation with IHC remains critical for accurate diagnosis and disease mapping. Additionally, this case illustrates the utility of MMS with IHC to achieve complete margin control in recurrent and anatomically complex disease. Ultimately, recognition of ectopic EMPD and incorporation of margin-controlled excision techniques are important in optimizing diagnostic accuracy, reducing recurrence risk, and improving patient outcomes.

### Declaration of generative AI and AI-assisted technologies in the writing process

None.

## Conflicts of interest

None disclosed.
